# Trait representation of embodied cognition in dancers pivoting on the extended mirror neuron system: a resting-state fMRI study

**DOI:** 10.3389/fnhum.2023.1173993

**Published:** 2023-07-10

**Authors:** Ching-Ju Yang, Hsin-Yen Yu, Tzu-Yi Hong, Chung-Heng Shih, Tzu-Chen Yeh, Li-Fen Chen, Jen-Chuen Hsieh

**Affiliations:** ^1^Institute of Brain Science, College of Medicine, National Yang Ming Chiao Tung University, Taipei City, Taiwan; ^2^Integrated Brain Research Unit, Division of Clinical Research, Department of Medical Research, Taipei Veterans General Hospital, Taipei City, Taiwan; ^3^Graduate Institute of Arts and Humanities Education, Taipei National University of the Arts, Taipei City, Taiwan; ^4^Department of Radiology, Taipei Veterans General Hospital, Taipei City, Taiwan; ^5^Institute of Biomedical Informatics, College of Medicine, National Yang Ming Chiao Tung University, Taipei City, Taiwan; ^6^Brain Research Center, National Yang Ming Chiao Tung University, Taipei City, Taiwan; ^7^Department of Biological Science and Technology, College of Biological Science and Technology, National Yang Ming Chiao Tung University, Hsinchu, Taiwan; ^8^Center for Intelligent Drug Systems and Smart Bio-devices (IDS^2^B), National Yang Ming Chiao Tung University, Hsinchu, Taiwan

**Keywords:** dancer, long-term training effect, embodied cognition, extended mirror neuron system, neuroplasticity, resting-state functional MRI, functional connectivity

## Abstract

**Introduction:**

Dance is an art form that integrates the body and mind through movement. Dancers develop exceptional physical and mental abilities that involve various neurocognitive processes linked to embodied cognition. We propose that dancers’ primary trait representation is movement-actuated and relies on the extended mirror neuron system (eMNS).

**Methods:**

A total of 29 dancers and 28 non-dancer controls were recruited. A hierarchical approach of intra-regional and inter-regional functional connectivity (FC) analysis was adopted to probe trait-like neurodynamics within and between regions in the eMNS during rest. Correlation analyses were employed to examine the associations between dance training, creativity, and the FC within and between different brain regions.

**Results:**

Within the eMNS, dancers exhibited increased intra-regional FC in various brain regions compared to non-dancers. These regions include the left inferior frontal gyrus, left ventral premotor cortex, left anterior insula, left posterior cerebellum (crus II), and bilateral basal ganglia (putamen and globus pallidus). Dancers also exhibited greater intrinsic inter-regional FC between the cerebellum and the core/limbic mirror areas within the eMNS. In dancers, there was a negative correlation observed between practice intensity and the intrinsic FC within the eMNS involving the cerebellum and basal ganglia. Additionally, FCs from the basal ganglia to the dorsolateral prefrontal cortex were found to be negatively correlated with originality in dancers.

**Discussion:**

Our results highlight the proficient communication within the cortical-subcortical hierarchy of the eMNS in dancers, linked to the automaticity and cognitive-motor interactions acquired through training. Altered functional couplings in the eMNS can be regarded as a unique neural signature specific to virtuoso dancers, which might predispose them for skilled dancing performance, perception, and creation.

## 1. Introduction

Dance is a multi-faceted art form that expresses ideas, emotions, and transformative thought processes through spatially and temporally coordinated actions (Sevdalis and Keller, 2011; Lin et al., 2013). These actions often involve dynamic interactions with other dancers. Based on the principles of embodied cognition theory, which asserts that cognitive processes are rooted in physical experiences, the mental reenactment of bodily actions during self-other interactions plays a vital role in higher-level cognition (Barsalou, 2008). The concept of embodied cognition holds significant relevance in the realm of dance training and offers a foundation for advancing our understanding of the ways in which perceptual, sensorimotor, and multisensory approaches support and promote learning (Sevdalis and Keller, 2011; Bläsing et al., 2012; Macrine and Fugate, 2020). Through training, accomplished dancers cultivate embodied representations of the body and movement that are founded on experiential knowledge (Bläsing and Schack, 2012). Skill learning in dance involves the enhancement of both motor and cognitive abilities resulting from continuous physical and mental practice. Dancers develop superior physical and mental abilities by engaging in embodied cognition, including motor control, attentional focus, sequence learning and memory, visuomotor imagery, action and intention understanding, emotion understanding, aesthetic creation and expression, and social cohesion (Sevdalis and Keller, 2011; Bläsing et al., 2012; Christensen et al., 2017). It is notable that skill learning in dance also elicits visual creativity (Fink et al., 2009; Fink and Woschnjak, 2011). Dancers are skilled in motor simulation and imagery to mentally rehearse new movements for creative artistic expressions (Nordin and Cumming, 2005; Bläsing et al., 2012). Embodied-based skill learning in dance enables the motor system to facilitate movement creation and engages higher-level cognitive areas for creative processes (Gonen-Yaacovi et al., 2013; Boccia et al., 2015). In other words, dance is an art form that integrates the body and mind through low-level perception-action coupling and high-level cognitive processes.

Intensive dance practice over several years may promote neurocognitive functioning on multiple levels, inducing persistent alterations in the brain and mind, commonly referred to as trait effects, that endure over an extended period of time (Cahn and Polich, 2006). This repeated practice leads to functional and structural changes within the motor system, specifically the sensorimotor network (which includes the postcentral gyrus, precentral gyrus, supplementary motor area, premotor cortex, and putamen) and the cortico-basal ganglia loops (which are functionally connected to the middle cingulate cortex and putamen), resulting in their motor expertise (Hanggi et al., 2010; Karpati et al., 2015; Li et al., 2015; Lu et al., 2018). Furthermore, dancers possess the capacity to be keen observers to dance moves due to their heightened perceptual sensitivity resulting from their visual and motor exposure (Calvo-Merino et al., 2010). Variances in brain activity between experienced and inexperienced dancers when observing dance suggest that dance experience influences perception and internal motor simulation (Calvo-Merino et al., 2005). The action observation network (AON), which is responsible for visual analysis of action, visuomotor skills, and sequence learning, is highly engaged in expert dancers (Calvo-Merino et al., 2005; Cross et al., 2006, 2009; Burzynska et al., 2017). The core regions of the AON, including the inferior frontal gyrus (IFG), ventral premotor cortex (PMv), and inferior parietal lobule (IPL), exhibit mirror properties similar to those of the mirror neuron system (MNS) and are active during both action execution and observation (Caspers et al., 2010; Molenberghs et al., 2012). The MNS, which couples action and perception to facilitate an understanding of others’ actions through motor simulation (Rizzolatti et al., 2001), is distributed throughout the brain and can be categorized into core MNS (including the PMv, IFG, and IPL) and limbic MNS (including the insula and anterior mesial frontal cortex) (Pineda, 2008; Cattaneo and Rizzolatti, 2009; Ramsey et al., 2021). In addition, an extended cortico-subcortical MNS involving subcortical processing associated with cortico-cerebellar and cortico-basal ganglia loops has been proposed by some researchers (Caligiore et al., 2013; Bonini, 2017). However, it has been a subject of ongoing debate whether the motor system has an influence on perception (Hickok et al., 2011).

We reasoned that the neural mechanisms underlying embodied cognition of skilled dancers are primarily movement-actuated and involve regions with mirror properties, including the IFG, PMv, IPL, intraparietal sulcus (IPS), insula, anterior cingulate cortex (ACC), supplementary motor area (SMA), sensorimotor cortex, primary motor cortex (M1), superior temporal sulcus (STS), dorsal premotor cortex (PMd), middle temporal gyrus (MTG), superior parietal lobule (SPL), cerebellum, and basal ganglia (Pineda, 2008; Molenberghs et al., 2012; Bonini, 2017; Bonini et al., 2022). Collectively, these regions constitute the so-called extended MNS (eMNS) (Pineda, 2008; Cattaneo and Rizzolatti, 2009; Molenberghs et al., 2012; Bonini, 2017; Ramsey et al., 2021), which we suggest as the neural network underlying the embodied cognition of skilled dancers. The eMNS may encompass neural characteristics associated with the consolidation of dance experiences. The eMNS, which includes various regions involved in the core MNS, limbic MNS, AON, cortico-cerebellar loops, and cortico-basal ganglia loops (Rizzolatti et al., 2001; Cattaneo and Rizzolatti, 2009; Caligiore et al., 2013; Turella et al., 2013; Bonini, 2017; Ramsey et al., 2021), may undergo changes in intrinsic functional connectivity (FC) as a result of long-term dance training. These alterations in neural systems related to motor representations (such as execution, observation, simulation, and imagery processes) as well as higher-level cognitive and socioemotional functionality could collectively support dancers in complex cognitive-motor and social interactions.

Given the extensive and dedicated practice involving various cognitive and psychological processes, it is reasonable to propose that prolonged dance training significantly impacts how the brain functions in perceiving and performing dance. We accordingly hypothesized that the neural representation of dance expertise would exhibit a complex and optimized pattern of connectivity. To explore this, we utilized resting-state functional magnetic resonance imaging (fMRI) and examined brain-behavior correlations to understand the organization of the dancer’s brain and its connection to their dance experience. Our goal was not to focus on specific cognitive or mental processes during a particular task, but rather to gain insight into the overall associations between the dancer’s brain and their dance expertise. In essence, we aimed to understand the overall impact of long-term dance training on brain connectivity patterns, rather than establishing a direct correspondence between specific cognitive processes and brain activation. Resting-state FC has been shown to be modulated by long-term experiences such as cognitive training, motor skill learning, and art learning (Lewis et al., 2009; Dayan and Cohen, 2011; Lin et al., 2013). We sought to identify the important regions, which are critical for efficient neural information transfer within and between regions in dancers’ brains during rest (trait representation). Key regions in dancers’ brains were identified using regional homogeneity (ReHo) analysis to measure local synchronization in resting brain activity and characterize local communication within a functionally segregated region (Zang et al., 2004; Yan et al., 2013; Deng et al., 2016). Seed-based FC analysis was then performed on regions identified as significant by ReHo analysis to examine long-distance communication patterns across functionally segregated regions in dancers. We hypothesized that dancers demonstrate distinct neuroplasticity in both intra- and inter-regional FC within the eMNS, influenced by their immersive embodied learning experiences. Importantly, this trait effect persists even during the non-dancing (resting) state, representing the consolidated neural manifestation of dance expertise, rather than being limited to the periods during or immediately after dancing.

## 2. Materials and methods

### 2.1. Participants

We recruited 29 dance students (dancer group: DANCE; 23.14 ± 2.95 years; 7 males) from art universities and 28 non-dancer controls (control group: CON; 22.79 ± 2.69 years; 4 males). This study was part of an integrated multimodal neuroimaging research program focusing on undergraduate and graduate students majoring in various forms of arts. Both groups were matched for age and general education. DANCE reported 14.52 ± 4.52 years of dance training with 28.76 ± 12.81 h of weekly practice. Among the dance students included in the study, seven of them had undergone music lessons, and one student had additional training in visual arts. CON had less than 3 years of institutional dance or music training and were not currently practicing. None of the participants were skilled athletes or reported a history of neuropsychiatric disorder, and all were right-handed, as assessed using the Edinburgh Handedness Inventory (Oldfield, 1971). The study was approved by the Institutional Review Board of Taipei Veterans General Hospital and written informed consent was obtained from each participant. Demographic information and dance training details are provided in Table 1.

**TABLE 1:** Demographic characteristics and psychological results.

	DANCE (*n* = 29)	CON (*n* = 28)	*p*-value
Sex (male/female)	7/22	4/24	0.346
Age (years)	23.14 ± 2.90	22.79 ± 1.64	0.574
Education (years)	16.07 ± 1.19	16.11 ± 1.20	0.904
Onset of dance training (years)	8.62 ± 4.07	–	–
Duration of dance training (years)	14.52 ± 4.52	–	–
Weekly practice (hours)	28.76 ± 12.81	–	–
Daily practice (hours)	4.88 ± 2.23	–	–
**ATTA**			
CI	66.93 ± 6.54	64.46 ± 6.30	0.153
Fluency	14.97 ± 1.97	14.89 ± 1.69	0.882
Originality	17.17 ± 1.77	15.32 ± 2.51	0.002**
Elaboration	16.00 ± 1.44	16.04 ± 2.36	0.946
Flexibility	14.90 ± 1.93	14.36 ± 1.54	0.251
Verbal creativity	0.72 ± 0.75	0.64 ± 0.83	0.699
Visual creativity	3.17 ± 1.73	3.21 ± 2.20	0.937

Data are expressed as mean ± standard deviation.

**p < 0.01. ATTA, Abbreviated Torrance Test for Adults; CI, creativity index.

### 2.2. Psychological measurement

Our integrated multimodal neuroimaging research program collected psychological data to portray demographic and psychological characteristics of young Taiwanese artists (20–30 years). In this study, we used the Abbreviated Torrance Test for Adults (ATTA) to assess general creativity and verbal/non-verbal creative thinking (Chen, 2006). The employed ATTA battery includes one verbal and two figural tests, with four norm-referenced creativity indicators (fluency, originality, elaboration, flexibility), a creativity index (CI; the sum of the aforementioned 4 measures), and two criterion-referenced creativity indicators (verbal and visual creativity) calculated for an overall creativity profile of each participant (Kharkhurin and Samadpour Motalleebi, 2008; Althuizen et al., 2010; Kharkhurin, 2010; Shen and Lai, 2014; Sunavsky and Poppenk, 2020). We compared the creativity profile of DANCE and CON, based on the six indicators of general creativity. Between-group differences were analyzed using a two-sample *t*-test (SPSS Statistics version 22.0, SPSS Inc., USA) with a significance level of *p* < 0.05.

### 2.3. MRI data acquisition

Resting-state fMRI data were obtained using the 3T MAGNETOM Trio™ system located at the National Yang-Ming University. Participants were positioned supine in the scanner with foam cushions holding their heads fixed to reduce motion artifacts. T2*-weighted gradient echo planar image (EPI) sequences were used to obtain resting-state fMRI images for each participant, with the following parameters: repetition time (TR) = 2500 ms, echo time (TE) = 30 ms, flip angle = 90°, field of view (FOV) = 220 mm × 220 mm, slice thickness = 3.4 mm, slice number = 40, matrix size = 64 × 64, tilted angle = 30°, and voxel size = 3.4 mm × 3.4 mm × 3.4 mm. To mitigate potential initial fMRI signal instability, the scanner automatically discarded the first three volumes of the scan, which served as dummy scans. Each resting-state fMRI time series comprised 200 volumes, with a duration of 500 s per time series. Additionally, high-resolution T1-weighted structural images were acquired using the magnetization prepared rapid gradient echo (MPRAGE) sequence, with the following parameters: TR = 2530 ms, TE = 3.03 ms, flip angle = 70°, FOV = 224 mm × 256 mm, matrix size = 224 × 256, slice thickness = 1 mm. Participants were instructed to remain still and awake with their eyes open and not to think about anything in particular. Fourteen participants dropped out, and 12 were excluded, resulting in a final sample of 29 dancers and 28 non-dancer controls for analysis.

### 2.4. Data preprocessing

The Data Processing Assistant for Resting-State fMRI (DPARSF) V4.5 Advanced Edition, which is based on the Data Processing and Analysis of Brain Imaging (DPABI) toolbox version 4.1 (Yan et al., 2016), with Statistical Parametric Mapping (SPM12) implemented in MATLAB R2018b (The Math Works, Inc., Natick, MA, USA), was used to preprocess the data. The following steps were performed: (1) slice timing correction, (2) realignment for head motion correction, with exclusion of participants with head motion exceeding 2 mm displacement or 2° rotation in any of the cardinal directions (x, y, z), (3) co-registration of T1-weighted images to the mean functional image using intra-subject spatial alignment, (4) segmentation of gray matter, white matter, and cerebrospinal fluid using the unified segmentation model, (5) nuisance regression using the Friston 24-parameter model (Friston et al., 1996) and default masks from SPM for regressing out head motion parameters and signals from white matter and cerebrospinal fluid, (6) spatial normalization to a study-specific Diffeomorphic Anatomical Registration Through Exponentiated Lie Algebra (DARTEL) template (Ashburner, 2007), which was transformed to the Montreal Neurological Institute (MNI) space, with image resampling to 3 mm isotropic voxels, and (7) temporal band-pass filtering (0.01 to 0.1 Hz) to reduce the effects of high-frequency noise and low-frequency drift. Global signal regression (GSR) was not performed because it has been shown to exaggerate negative correlations (Murphy et al., 2009; Weissenbacher et al., 2009) and distort between-group differences (Saad et al., 2012).

### 2.5. ReHo analysis

Individual ReHo maps were generated using Kendall’s coefficient of concordance (KCC) of the time series between each voxel and its 26 nearest neighbors, in a voxel-wise manner (Zang et al., 2004). Individual ReHo maps were transformed to *z*-score maps, and the resulting standardized ReHo maps were smoothed using a Gaussian kernel with a full-width at half-maximum (FWHM) of 6 mm, as suggested by previous studies (Tian et al., 2012; Zuo et al., 2013). Between-group comparisons were conducted using a two-sample *t*-test in SPM, with statistical significance set at uncorrected voxel levels of *p* < 0.001 and *p* < 0.005, followed by the family wise error (FWE)-corrected cluster level of *p* < 0.05.

### 2.6. ReHo-seeded FC analysis

The same preprocessing procedures as for ReHo analysis were followed for FC analysis, with the exception of spatial smoothing, which was performed using a 6-mm FWHM Gaussian kernel prior to the analysis. Individual FC maps were generated by computing Pearson’s correlation coefficients (*r*) between the seed and other regions of the brain. The seed regions of interest (ROIs) were derived from brain regions exhibiting significant ReHo differences between groups (Wu et al., 2016). These seed ROIs were created by masking the brain regions with significant ReHo differences using the WFU Pickatlas 3.0.5 (Maldjian et al., 2003) with automatic anatomical labeling (AAL) atlas (Tzourio-Mazoyer et al., 2002). The reference time course was obtained by averaging the time courses of all voxels within the ReHo-seeded region (seed ROI). The FC map was generated by calculating the correlation coefficient between the reference time course and the time course of each voxel. The *r*-value of each voxel was transformed to a *z*-value using Fisher’s *r*-to-*z* transformation to normalize the distribution. Between-group differences were examined using two-sample *t*-tests on ReHo-seeded FC maps of each seed, with significance set at uncorrected voxel levels of *p* < 0.001 and *p* < 0.005, followed by the FWE-corrected cluster level of *p* < 0.05 in SPM. Bonferroni’s correction was also performed for multiple comparisons by adjusting the *p*-value to 0.00625 (0.05 divided by 8), as eight seed ROIs were analyzed in this study.

### 2.7. Correlation analysis

In order to examine the effects of dance training on the brain, we conducted a correlation analysis between measures of ReHo and FC seeded by ReHo, with variables related to dance training and creativity indicators. The variables of dance training considered were the duration of training and the average daily/weekly practice time. Additionally, creativity indicators that showed significant differences between groups were included in the analysis. Regions that exhibited significant between-group differences in ReHo and ReHo-seeded FC were identified, and the extracted *z*-values from these regions were correlated with the dance training variables and creativity indicators. Statistical significance was defined as *p* < 0.05, and to account for multiple comparisons, a Bonferroni’s correction was applied by adjusting the *p*-value to 0.0125 (0.05 divided by 4), considering that four measures (training duration, daily practice, weekly practice, and originality) were analyzed.

## 3. Results

### 3.1. Demographic data and psychological results

There were no significant differences between groups in terms of demographic characteristics such as sex, age, or level of education. In terms of psychological characteristics, DANCE showed significantly higher scores for originality on the ATTA (DANCE: 17.17 ± 1.77, CON: 15.32 ± 2.51, *p* = 0.002), with no differences between groups in fluency, elaboration, flexibility, visual creativity, verbal creativity, or CI (Table 1).

### 3.2. Enhanced intra-regional FC within the eMNS in DANCE

**FIGURE 1:**
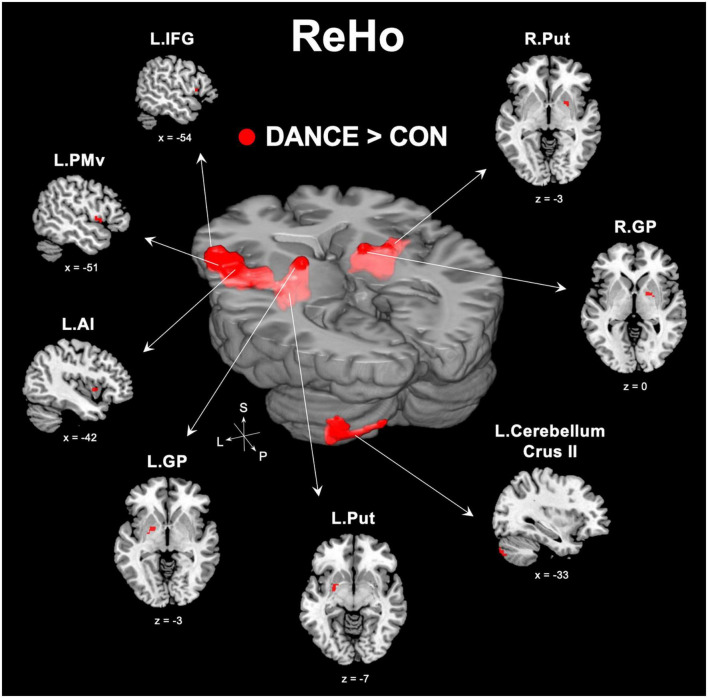
Between-group differences in ReHo. ReHo analysis confirmed the importance of functionally segregated regions, including the left IFG, left PMv, left AI, left cerebellum crus II, bilateral GP, and bilateral Put, within the eMNS in DANCE and the enhanced intra-regional integration of these distributed regions. The red color denotes ReHo regions in DANCE that differ significantly from those in CON. All figures are displayed according to the neurological convention. Details of the significant ReHo results are listed in Table 2. ReHo, regional homogeneity; IFG, inferior frontal gyrus; PMv, ventral premotor cortex; AI, anterior insula; GP, globus pallidus; Put, putamen; eMNS, extended mirror neuron system; L, left; R, right; S, superior; P, posterior.

We used ReHo to assess intra-regional functional integration/segregation across the whole brain to identify key regions of resting-state brain dynamics in dancers. The ReHo analysis showed significantly higher values in several regions, including the left IFG, left anterior insula (AI), left PMv, left cerebellum (crus II), and subregions of the bilateral basal ganglia (globus pallidus [GP] and putamen) in DANCE compared to CON (Figure 1 and Table 2). These regions were the key neural substrates within the eMNS. The results verified the significance of the eMNS in DANCE. The observed heightened local synchronization within these regions suggests enhanced efficiency in the intra-regional information flow within dancers’ eMNS.

**TABLE 2:** Between-group differences in ReHo.

Region	BA	MNI coordinates			*t-value*	Cluster size
		*x*	*y*	*z*		
**DANCE > CON**						
L IFG	44	−54	15	9	4.91*	245
L AI	13	−36	3	0	4.17*	
L PMv	6	−51	6	3	4.15*	
L GP		−18	0	−3	3.79	
L Putamen		−30	−15	−6	2.73	
L Cerebellum crus II		−33	−84	−42	4.42*	160
R GP		15	3	0	3.91	145
R Putamen		27	0	−3	3.91	
**DANCE < CON**						
NS						

IFG, inferior frontal gyrus; AI, anterior insula; PMv, ventral premotor cortex; GP, globus pallidus; BA, Brodmann’s area; MNI, Montreal Neurological Institute; L, left; R, right; NS, not significant. All results are reported at a family wise error (FWE)-corrected cluster threshold of p < 0.05 following an uncorrected voxel threshold of p < 0.005.

*Denotes significant ReHo results thresholded at an uncorrected voxel threshold of p < 0.001.

### 3.3. Enhanced inter-regional FC between core/limbic mirror areas and the posterior cerebellum in DANCE

**FIGURE 2:**
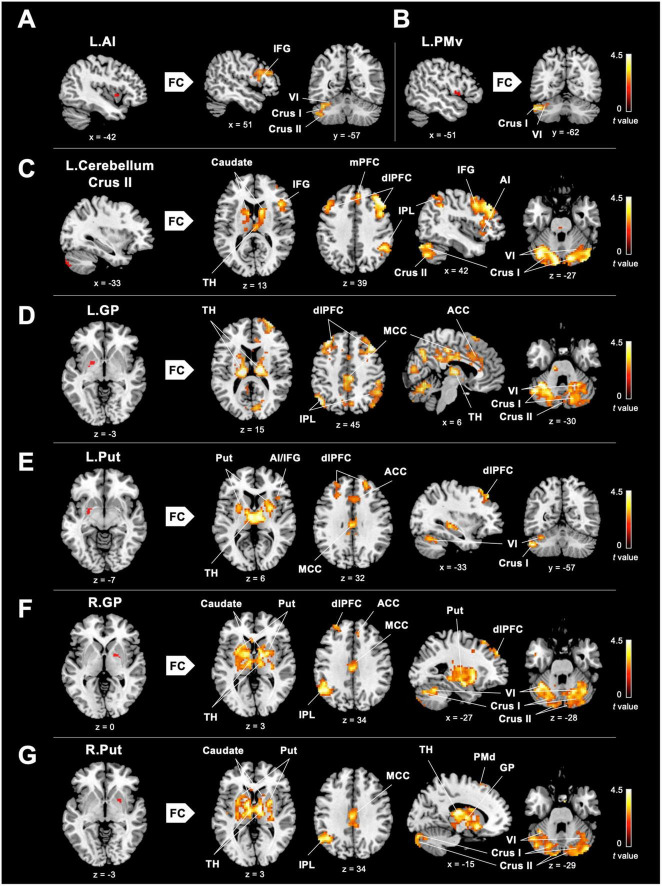
Between-group differences in ReHo-seeded FC. **(A,B)** DANCE presented greater inter-regional FCs between the core (the right IFG) and limbic mirror areas (the left AI), and between the core/limbic mirror areas (the left PMv/AI) and the left posterior cerebellum within the eMNS. **(C)** DANCE presented significantly elevated inter-regional FCs seeded from the left posterior cerebellum (crus II) to the bilateral posterior cerebellum, basal ganglia, dlPFC, and regions within the eMNS (the IFG, AI, IPL, and mPFC). **(D–G)** Inter-regional FCs seeded from the bilateral basal ganglia forming an integrated cerebello-basal ganglia-thalamo-cortical system, wherein resonance with the bilateral TH, posterior cerebellum, dlPFC, MCC, IPL, and ACC was more pronounced in DANCE than in CON. Details for the significant ReHo-seeded results are listed in Table 3. All figures are displayed according to the neurological convention. The red color denotes seed regions obtained from ReHo analysis. FC, functional connectivity; dlPFC, dorsolateral prefrontal cortex; mPFC, medial prefrontal cortex; IPL, inferior parietal lobule; TH, thalamus; MCC, middle cingulate cortex; ACC, anterior cingulate cortex; PMd, dorsal premotor cortex; also refer to Figure 1 for other abbreviations.

To investigate the role of the eMNS in dancers, we utilized seed-based FC analysis to examine FC patterns seeded from significant ReHo results. DANCE showed that the left AI and left PMv were both strongly connected to the left posterior cerebellum. Intrinsic FC between the posterior cerebellum and the core/limbic mirror areas within the eMNS was also consistently higher in DANCE compared to CON. However, no significant differences were observed in the FC seeded from the left IFG. These findings suggest a synergy between core/limbic mirror areas and the posterior cerebellum within the eMNS in DANCE. Details can be found in Table 3 and Figures 2A–C.

**TABLE 3:** Altered FC seeded from the ReHo results thresholded at an uncorrected voxel level of *p* < 0.001 in DANCE compared to CON.

Seed	Region	BA	MNI coordinates			*t*-value	Cluster size
			*x*	*y*	*z*		
**DANCE > CON**							
L IFG	NS						
L PMv	L Cerebellum crus I		−51	−63	−33	4.23	161
	L Cerebellum lobule VI		−36	−51	−30	4.06	
L AI	L Cerebellum crus I		−42	−60	−30	4.64*	256^†^
	L Cerebellum lobule VI		−36	−51	−30	3.63*	
	R IFG	45	51	33	21	3.65*	236^†^
	R IFG	44	51	18	21	3.46*	
L Cerebellum crus II	L Cerebellum crus II		−9	−81	−30	4.99*	1686^†^
	R Cerebellum crus I		15	−78	−24	4.94*	
	L Cerebellum crus I		−36	−72	−27	4.7*	
	R MFG (dlPFC)	9	39	30	39	4.54*	1846^†^
	R Caudate		12	6	15	4.27	
	R AI	13	39	18	−6	3.94	
	R MFG (PMd)	6	39	6	57	3.9*	
	R IFG	44	45	18	12	3.89*	
	R IPL	40	51	−45	39	4.12*	297^†^
	R ACC	32	9	39	30	4.04	315^†^
	R ITG	37	63	−57	−9	3.99	226
	R MTG	21	69	−42	3	2.81	

MNS, mirror neuron system; IFG, inferior frontal gyrus; AI, anterior insula; PMv, ventral premotor cortex; MFG, middle frontal gyrus; dlPFC, dorsolateral prefrontal cortex; PMd, dorsal premotor cortex; IPL, inferior parietal lobule; ACC, anterior cingulate cortex; ITG, inferior temporal gyrus; MTG, middle temporal gyrus; BA, Brodmann’s area; MNI, Montreal Neurological Institute; L, left; R, right; NS, not significant.

*Denotes significant results thresholded at an uncorrected voxel threshold of *p* < 0.001.

^†^Denotes significant cluster results after Bonferroni’s correction (*p* < 0.05/8).

### 3.4. Enhanced extended cortico-subcortical MNS in DANCE

Our hypothesis that dancers possess neuroplasticity, involving enhanced engagement of subcortical structures within the eMNS, was supported by our findings. Specifically, our findings showed that DANCE exhibited an elevated extended cortico-subcortical MNS compared to CON. These findings are consistent with our previous graph-based study that highlighted intra-modular connections within the cortico-cerebellar loops among the cerebellum, dorsolateral prefrontal cortex (dlPFC), IPL, and caudate in dancers (Lin et al., 2013).

In DANCE, enhanced FC was observed between the left posterior cerebellum (crus II) and the contralateral posterior cerebellum, bilateral dlPFC, and right IPL within the cortico-cerebellar loops (Figure 2C and Table 3). Furthermore, there was an observed FC between the posterior cerebellum (specifically lobule VI, crus I, and II) and the core/limbic mirror areas, as well as the basal ganglia, which was found to be stronger in DANCE compared to CON (Figures 2C–G and Table 4). In line with this, DANCE also presented elevated FCs seeded from the basal ganglia (GP and putamen) to the core/limbic mirror areas (IPL and ACC) within the eMNS (Figures 2D–G and Table 4). Moreover, DANCE showed enhanced FCs seeded from the basal ganglia (GP and putamen) in both the sensorimotor and cognitive/associative circuits of the cortico-basal ganglia loops, which were intrinsic connections of the basal ganglia to the GP, putamen, ventrolateral/ventroanterior thalamus, and PMd in the former circuit and to the dlPFC, mediodorsal thalamus, caudate, and GP in the latter circuit (Figures 2D–G and Table 4; Haber, 2003; Yin and Knowlton, 2006; Worbe et al., 2012; Macpherson and Hikida, 2019). These findings suggest that the intricate interplay among the cerebellum, basal ganglia, thalamus, and cortical areas can be viewed as a synergistic cortico-subcortical integration of the eMNS in dancers. This integration has been referred to as the extended cortico-subcortical MNS (Bonini, 2017).

**TABLE 4:** Altered FC seeded from the ReHo results thresholded at an uncorrected voxel level of *p* < 0.005 in DANCE compared to CON.

Seed	Region	BA	MNI coordinates			*t*-value	Cluster size
			*x*	*y*	*z*		
**DANCE > CON**							
L GP	L Cerebellum crus I		−48	−57	−39	6.2*	3612^†^
	R Cerebellum lobule VI		12	−69	−24	4.83*	
	L Cerebellum lobule VI		−36	−51	−30	4.77*	
	R Cerebellum crus II		12	−84	−39	4.38*	
	R Thalamus (VPL)		15	−18	15	5.42*	
	L Thalamus (VPL)		−15	−21	15	5.14*	
	R Putamen		27	12	−9	4.19	
	R MFG (dlPFC)	8	39	27	48	4.82*	2064^†^
	R MCC	23	3	−21	33	4.8*	
	R SFG (dlPFC)	9/46	24	45	24	4.36*	
	L MFG (dlPFC)	9	−33	36	45	4.95*	638^†^
	L MFG (PMd)	6	−18	12	66	3.33	
	R IPL	40	54	−48	51	4.07*	469^†^
	L IPS	39	−48	−66	45	5.12*	445^†^
L Putamen	L MFG (dlPFC)	8	−36	27	51	3.71	233^†^
	R Thalamus (VL)		15	−15	12	4.92*	1393^†^
	L Thalamus (LP)		−12	−21	15	4.71*	
	R Thalamus (MD)		6	−15	9	4.63*	
	R Putamen		27	3	0	4.6*	
	L Thalamus (VL)		−6	−12	6	4.52*	
	R GP		12	3	−6	4.41*	
	L Thalamus (MD)		−3	−18	9	4.31*	
	L Cerebellum crus I		−51	−57	−39	4.61	164
	L Cerebellum lobule VI		−36	−51	−30	3.61	
	R SFG (dlPFC)	9/46	24	45	24	4.27	185
	R ACC	32	3	24	30	3.4	190
R GP	R Thalamus (VA)		12	−3	3	5.05*	1723^†^
	L Putamen		−27	9	−3	4.42*	
	L Caudate/NAc		−12	12	−9	4.32*	
	L Thalamus (VL)		−6	−12	6	4.09*	
	R Thalamus (VPL)		12	−18	6	4.05*	
	L Cerebellum crus I		−48	−57	−39	4.79*	1569^†^
	L Cerebellum lobule VI		−36	−51	−30	4.52*	
	R Cerebellum lobule VI		21	−60	−21	4.49*	
	R Cerebellum crus I		45	−78	−33	4.38*	
	L MCC	23	−3	−21	33	3.91	224^†^
	L MFG (dlPFC)	8	−36	27	48	3.41	280^†^
	L MFG (PMd)	6	−21	15	60	3.45	
	L IPS	39	−45	−63	45	4.71*	376^†^
R Putamen	R Thalamus (VA)		9	−6	3	5.01*	2151^†^
	L Thalamus (LP)		−12	−21	15	4.93*	
	R Thalamus (VL)		9	−12	6	4.73*	
	L Thalamus (VL)		−6	−12	6	4.63*	
	L Caudate/NAc		−12	12	−9	4.25*	
	L Cerebellum lobule VI		−36	−51	−27	4.57*	606^†^
	L Cerebellum crus I		−51	−57	−39	4.52*	
	R Cerebellum crus I		42	−78	−33	4.21*	503^†^
	L MFG (dlPFC)	8	−39	21	48	3.17	151
	L SFG (PMd)	6	−18	21	63	3.09	
	L IPS	39	−45	−63	45	5.06*	335^†^

MNS, mirror neuron system; GP, globus pallidus; VPL, ventroposterolateral; MFG, middle frontal gyrus; dlPFC, dorsolateral prefrontal cortex; MCC, middle cingulate cortex; SFG, superior frontal gyrus; IPS, intraparietal sulcus; PMd, dorsal premotor cortex; IPL, inferior parietal lobule; VL, ventrolateral; LP, lateroposterior; MD, mediodorsal; ACC, anterior cingulate cortex; VA, ventroanterior; NAc, nucleus accumbens; BA, Brodmann’s area; MNI, Montreal Neurological Institute; L, left; R, right.

*Denotes significant results thresholded at an uncorrected voxel threshold of *p* < 0.001.

^†^Denotes significant cluster results after Bonferroni’s correction (*p* < 0.05/8).

### 3.5. Correlations between functional connectivity and behavioral variables

**FIGURE 3:**
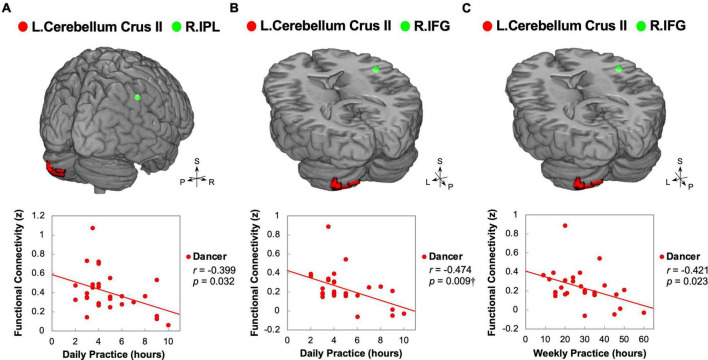
Correlations between daily/weekly dance practice and functional connectivity linking the posterior cerebellum and right core MNS in dancers. DANCE revealed significant associations between the frequency of daily or weekly dance practice and FC between the posterior cerebellum and the right core MNS. Specifically, the FC between the left posterior cerebellum (crus II) and the right IPL exhibited a negative correlation with the average weekly practice time in DANCE **(A)**. Additionally, the FC between the left posterior cerebellum (crus II) and the right IFG showed negative correlations with both the average daily and weekly practice time in DANCE **(B,C)**. In the figures, the seed regions obtained from ReHo analysis and the FC target regions are represented by red and green colors, respectively. Please refer to Figures 1, 2 for abbreviation details. ^†^Denotes significant results after Bonferroni’s correction (*p* < 0.0125).

**FIGURE 4:**
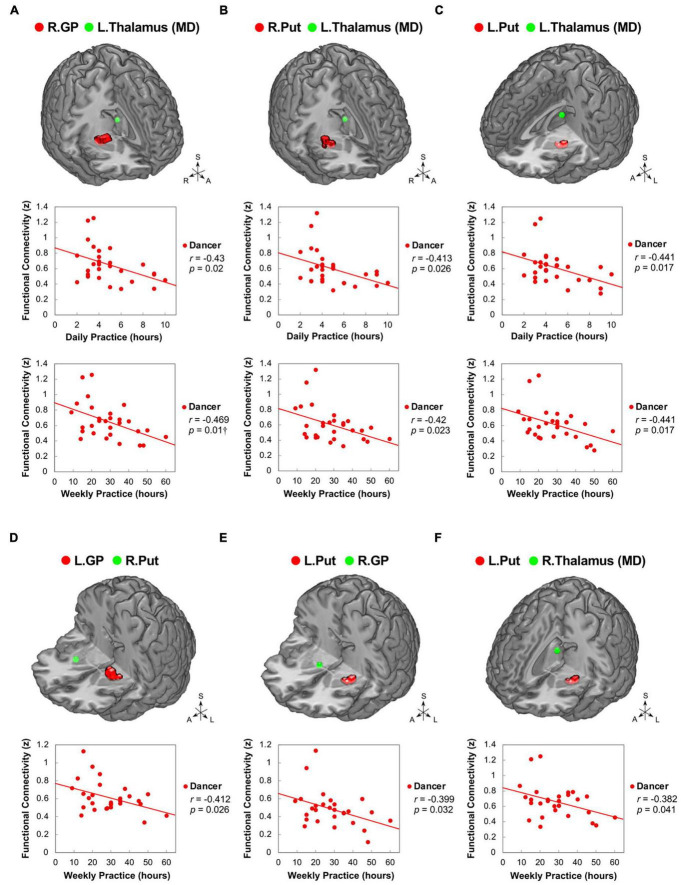
Correlations between daily/weekly dance practice and functional connectivity within the cortico-basal ganglia loops in dancers. DANCE revealed significant correlations between daily/weekly dance practice and FC within the cortico-basal ganglia loops. Specifically, FC between the right GP and left thalamus (MD) **(A)**, as well as bilateral Put and left thalamus (MD) **(B,C)**, showed negative correlations with the average daily and weekly practice time in DANCE. Furthermore, FC between the left GP and right Put **(D)**, left Put and right GP **(E)**, and left Put and right thalamus (MD) **(F)** exhibited negative correlations with the average weekly practice time in DANCE. The seed regions obtained from ReHo analysis and FC target regions are represented by red and green colors, respectively. MD, mediodorsal; A, anterior; also refer to Figures 1, 2 for other abbreviations. ^†^Denotes significant results after Bonferroni’s correction (*p* < 0.0125).

Our study aimed to examine the associations between ReHo and ReHo-seeded FC and demographic/psychological characteristics, including dance training variables and creativity indicators. The objective was to gain a better understanding of the overall connections between the dancers’ brains and their expertise in dance. Pearson’s correlation analysis revealed that DANCE showed significantly negative correlations between the average practice time per day/week and FC, but no clear correlation between the duration of dance training and FC. These findings suggest that the intensity of training is more strongly associated with spontaneous brain dynamics than the duration of training. Our results were supported by the negative correlations between the average practice time in DANCE and functional couplings between the posterior cerebellum and the right core MNS (IFG and IPL) (Figures 3A–C), as well as between the key regions of the sensorimotor and cognitive/associative cortico-basal ganglia circuits (the right GP–left mediodorsal thalamus, right putamen–left mediodorsal thalamus, left putamen–bilateral mediodorsal thalamus, left GP–right putamen, and left putamen–right GP FCs) (Figures 4A–F). These findings indicate a link between the intensity of dance training and FC in the eMNS of dancers.

**FIGURE 5:**
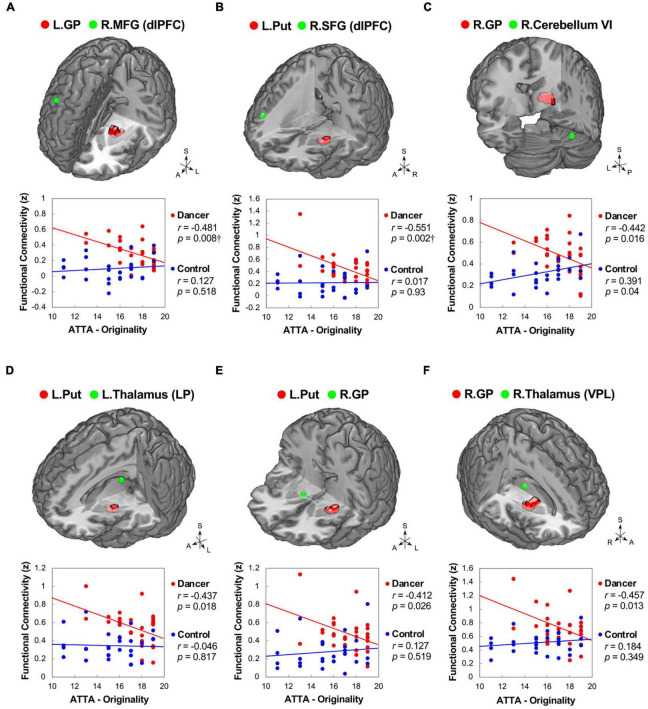
Correlations between originality and functional connectivity within the integrated cerebello-basal ganglia-thalamo-cortical system in dancers. DANCE revealed significant correlations between originality and FC within the integrated cerebello-basal ganglia-thalamo-cortical system. Negative correlations were observed between originality scores in DANCE and FCs within the system, including left GP-right dlPFC **(A)**, left Put-right dlPFC **(B)**, right GP-right posterior cerebellum (lobule VI) **(C)**, left Put-left thalamus (LP) **(D)**, left Put-right GP **(E)**, and right GP-right thalamus (VPL) **(F)** (depicted in red). It is worth noting that, except for a significantly positive correlation between originality scores and FC between the right GP and right posterior cerebellum (lobule VI), no significant relationship was observed between originality scores and FCs within the integrated cerebello-basal ganglia-thalamo-cortical system in CON (depicted in blue). The seed regions obtained from ReHo analysis and FC target regions are represented by red and green colors, respectively, in the three-dimensional brain. LP, lateroposterior; VPL, ventroposterolateral; MFG, middle frontal gyrus; SFG, superior frontal gyrus; ATTA, Abbreviated Torrance Test for Adults; A, anterior; also refer to Figures 1, 2 for other abbreviations. ^†^Denotes significant results after Bonferroni’s correction (*p* < 0.0125).

In DANCE, the originality scores were negatively correlated with functional couplings between sensorimotor and cognitive/associative regions in the cortico-cerebellar-basal ganglia system, such as the left GP–right dlPFC (BA 8), left putamen–right dlPFC (BA 9/46), and right GP–right posterior cerebellum (lobule VI) FCs (Figures 5A–C). Additionally, we found negative correlations between originality scores and intrinsic FCs within the cortico-basal ganglia loops, such as the left putamen–left lateroposterior thalamus, left putamen–right GP, and right GP–right ventroposterolateral thalamus FCs in DANCE (Figures 5A–F). These findings suggest that originality in dancers may be related to functional connections between regions involved in sensorimotor and cognitive/associative processing.

There were no notable differences between groups concerning the association between demographic/psychological characteristics and ReHo.

## 4. Discussion

In our ReHo and ReHo-seeded FC analyses, we found virtuoso-specific neural signature that manifest embodied cognition as increased functional connections within the eMNS in DANCE. Our findings suggest that the movement-actuated synergy between distributed brain areas within the eMNS is critical for expert dancers.

### 4.1. Trait representation in the eMNS underpinning embodied simulation-driven mirroring in dancers

The current study observed a left-lateralized increase in ReHo within the eMNS (including IFG, AI, and PMv) among dancers, suggesting neuroplasticity associated with learning. The dominance of the left hemisphere in the eMNS of dancers could be attributed to extensive dance practice, as the left hemisphere is known for its role in controlling well-established behavior in familiar situations (Mutha et al., 2012; Sainburg, 2016). The intricate interplay between the brain, body, and environment shapes the intrinsic functional architecture of dancers’ eMNS, with context and experience playing significant roles (Clark, 2016).

The left IFG and adjacent left PMv in the core MNS of dancers appear to exhibit functional segregation, as suggested by between-group results of FCs seeded from those two areas. In the case of the IFG of dancers, enhanced ReHo with no ReHo-seeded FC was observed in this region, implying local functional integration with functional specialization (Table 3; Dong et al., 2014; Wu et al., 2016). It is possible that intra-regional hyperconnectivity in the left IFG may be a result of the pattern perception of the sequence of dance steps during dance training, enhancing dancers’ action understanding of dance choreographies and reinforcing the notion that dance is a non-verbal language expressed through gestural movements (Udden and Bahlmann, 2012; Bachrach et al., 2016). The left ventral posterior IFG (BA 44) is associated with language-related lateralization but may also play a role in non-language related motor behavior in dancers, particularly in imitative behavior for coding the goal of action (Iacoboni, 2005; Molnar-Szakacs et al., 2005; Zardi et al., 2021). On the other hand, we observed increased ReHo in the left PMv in dancers (Figure 1), indicating a strengthened coupling between action and perception as a result of dance training. This enhanced intra-regional connectivity suggests an improved ability to understand actions performed by others (Sevdalis and Keller, 2011; Novembre and Keller, 2014).

Our findings reveal not only enhanced connectivity within the core MNS but also heightened connectivity within the limbic MNS, located between the left dorsal and left ventral AI. The dorsal and ventral AI regions are involved in higher-level cognitive functions like switching, inhibition, and error processing, as well as affective processes related to emotions (Uddin et al., 2014). For dancers, the AI may act as a critical bridge linking action representation to both cognitive and emotional aspects (Carr et al., 2003; Luo et al., 2014; Gujing et al., 2019). Additionally, we observed strong inter-regional connections originating from the left AI toward the posterior cerebellum (crus I and lobule VI) and the right IFG. This suggests that dancers possess an elevated sense of self-other awareness and an enhanced ability to detect salient information during the observation and imitation of dance movements (Carr et al., 2003; Walther et al., 2011; Gujing et al., 2019). Overall, our findings suggest that the integration within and between mirror areas contributes to the mechanisms of embodied simulation and mirroring, which are fundamental for understanding actions and emotions.

The observed enhanced intra-regional FC patterns within core/limbic mirror areas can be interpreted as a result of experience-dependent neuroplasticity that arises from embodied learning in dancers. These patterns reflect not only a transient neural state but a persistent neural trait associated with the consolidation of experience. In traditional terms, “consolidation” refers to the stabilization of memory traces. Within the framework of system memory consolidation (Lewis et al., 2009), the Hebbian rule of synaptic plasticity plays a crucial role and has been extensively studied in various fields, including physiology, psychophysics, and computation (Goto, 2022). Building upon the principles of Hebbian learning, which include Hebbian pruning, patterns of activity and connectivity during the learning process can shape the resting-state functional connectivity of experts (Lewis et al., 2009; Harmelech et al., 2013; Kim and Knosche, 2017).

Our neuroimaging findings of enhanced intra-regional functional connectivity within core/limbic mirror areas in dancers are in line with previous research that underscores the significance of the MNS in artistic training, specifically dance (Lin et al., 2013). Additionally, studies have highlighted the role of the MNS in imitative and embodied learning (Macrine and Fugate, 2021; Butera and Aziz-Zadeh, 2022). The MNS comprises neurons that not only activate during the execution of an action but also during the observation of others performing the same action (Rizzolatti and Craighero, 2004). This system plays a pivotal role in action understanding and imitation, suggesting that dancers’ cognitive abilities may be contingent upon the reenactment of sensory and motor representations following extensive training (Caramazza et al., 2014). It is commonly observed that dancers exhibit mirror-like activity when observing intricate dance movements (Calvo-Merino et al., 2005; Cross et al., 2006), implying that mirror mechanisms are crucial for experiential motor learning. According to the theories of embodied cognition (Caramazza et al., 2014; Keysers et al., 2018) and embodied learning (Fugate et al., 2018; Macrine and Fugate, 2021), these mechanisms potentially allow dancers to seamlessly incorporate observed actions into their own motor repertoire through the imitation and simulation of dance movements.

### 4.2. Embodied simulation and the internal forward models

Our study has shown that the enhanced functional connection between the cerebellum and mirror areas in dancers can be attributed to the combination of embodied simulation-driven mirroring mechanisms and predictive internal forward models. This connection connotes the integration of observed actions with an individual’s motor repertoire in the core mirror areas, which send a command to the cerebellum, which in turn sends a forecast back to the premotor area for subsequent action planning (Miall, 2003; Yarrow et al., 2009). The heightened FC between the left PMv and the left posterior cerebellum can be indicative of the essential role of cerebellar-premotor closed loop circuits in predicting the immediate consequences of intended dance movements and refining motor control (Figure 2B; Miall, 2003; Caligiore et al., 2013).

Our study also found that the left posterior cerebellum works in tandem with the core/limbic mirror areas, including the right IFG, right IPL, bilateral AI, and right ACC (Figures 2A, C), to process information related to the difference between actual and predicted sensorimotor feedback during or after a dance performance in an error-minimizing way (Koziol et al., 2012; Siman-Tov et al., 2019). By detecting bottom-up salience and prediction errors with these core/limbic mirror areas (Christoff et al., 2016), the cerebellum helps to update internal models and action plans for optimal dance performance in dancers (Levy and Wagner, 2011; Siman-Tov et al., 2019). Our results further support this notion by demonstrating a negative correlation between average practice time and left cerebellum-seeded functional connection to the right core MNS (IFG and IPL) in DANCE (Figure 3). It has previously been reported that the left cerebellum is associated with visuospatial and attention processing, and the right hemisphere is involved in detecting and responding to unexpected stimuli for updating ongoing actions (Mutha et al., 2012; Sainburg, 2016; Starowicz-Filip et al., 2021). These results suggest that the cerebellar connection with the right core MNS contributes to dancers’ ability to detect and respond to salient cues, update internal models, and optimize their actions for better dance performance. With increased practice time, dancers are more efficient in transferring information within internal models to advance embodied simulation (Miall, 2003; Caligiore et al., 2013).

The left posterior cerebellum (crus II) may play a role in learning action sequences linked to social traits from a social perspective (Pu et al., 2020). Given the strong link between the core/limbic mirror areas and the posterior cerebellum in DANCE, it is possible that the transfer of information within internal models can also be applied to social interactions based on embodied simulation, allowing dancers to easily comprehend the intentions behind the actions of others (Cattaneo and Rizzolatti, 2009; Zardi et al., 2021). Our results suggest that dance training aims to improve movement accuracy and optimize action planning, as well as to enhance the ability to mentally simulate actions and infer the intentions underlying choreographed movements (Miall, 2003; Wolpert et al., 2003; Karlinsky et al., 2017). We propose that dance learning progresses from observational motor learning to higher-level sociocognitive learning, facilitated by the continuous refinement and expansion of embodied action simulation, prediction, and planning.

### 4.3. Cortical-subcortical hierarchical organization in dancers

Our study found that dancers have stronger functional integration in cortico-basal ganglia and cortico-cerebellar loops compared to non-dancers, as evidenced by increased ReHo-seeded FCs. The cerebellum and basal ganglia, essential neural substrates involved in subcortical loops responsible for processing motor, cognitive, and emotional information, are believed to contribute to the development of action understanding abilities (Caligiore et al., 2013). The posterior cerebellum (crus II) in DANCE is connected to regions linked to the executive control networks, implying that it may facilitate higher-level cognitive processes and contribute to parallel cortico-cerebellar loops (Habas et al., 2009). Meanwhile, the basal ganglia are involved in sensorimotor, cognitive/associative, and limbic circuits in parallel within the cortico-basal ganglia loops that mediate motor, cognitive, and emotional functions (Haber, 2003; Lehericy et al., 2005; Yin and Knowlton, 2006).

It has been suggested that both the cortico-cerebellar and cortico-basal ganglia loops are involved in motor skill development (Hikosaka et al., 2002; Doyon et al., 2003). The GP is engaged in all functional circuits within the cortico-basal ganglia loop, while the putamen is mainly involved in the sensorimotor circuit. Both the execution and observation of manipulative actions activate the basal ganglia, thalamus, and cerebellum (Errante and Fogassi, 2020). Our findings related to the subcortical functional connections of the GP, putamen, and cerebellum support the assertion that the basal ganglia and cerebellum are important neural substrates for motor information transfer in dancers. These findings are consistent with those of previous studies on motor learning and dance (Doyon et al., 2009; Hardwick et al., 2013; Li et al., 2015). However, our findings on intra-regional FC of the basal ganglia and cerebellum at rest differ slightly from previous findings related to the putamen and anterior cerebellum during a simple dance task (Figure 1; Brown et al., 2006). Nevertheless, our findings indicate that the basal ganglia and cerebellum are engaged during dancing as well as in a resting state.

Our results showed that dancers’ subcortical areas, including the cerebellum, basal ganglia, and thalamus, as well as cortical areas, including the dlPFC, IPL, and PMd, interconnect functionally to form the integrated network/system of the cortico-cerebellar-basal ganglia system (Figures 2C–G), that is the extended cortico-subcortical MNS (Caligiore et al., 2017, 2019). This system plays a role in domain-general prediction across various functions such as somatic and visceral motor control, attention, timing, and social cognition (Siman-Tov et al., 2019). Specifically, enhanced cerebellar connectivity with the premotor and non-motor associative cortices in dancers facilitates cognitive-motor interactions, while the basal ganglia (caudate and nucleus accumbens) contribute to cognitive associative learning to assist in selecting a motor plan for goal-directed action (Grossberg, 2016). Our findings suggest that long-term dance training shapes the extended cortico-subcortical MNS and primes dancers for their goal-directed action, with the enhanced functional couplings in this system being the virtuoso-specific neural signature of intense physical and mental practice (Grossberg, 2016; Caligiore et al., 2017, 2019; Siman-Tov et al., 2019).

### 4.4. Identifying the contributions of cortical-subcortical hierarchical organization to cognitive-motor interaction and automaticity following intensive dance practice

The current study contributes to the growing body of evidence indicating that intensive dance practice leads to the integration of cortical-subcortical hierarchical organization, which is involved in cognitive-motor interactions. Compelling evidence has indicated that there are inverted U-shaped relationships between brain factors (such as brain activity and functional connectivity) and behavioral measures across different skill levels (i.e., expert, intermediate, and novice) (Brefczynski-Lewis et al., 2007; Chen et al., 2020, 2022). It is plausible that the functional connections within the subcortical regions associated with skill development undergo an initial increase followed by a subsequent decline as practice intensifies. This decrease in subcortical functional connections observed with prolonged practice may indicate neuroadaptation in cognitive-motor interactions. In dancers, these neural changes may follow a downward trajectory resembling an inverted U-shaped pattern. Our research findings support this idea, as we discovered negative correlations between practice time and functional connections (GP-putamen, putamen-GP, GP-thalamus, and putamen-thalamus FCs) in the cortico-basal ganglia loops (see Figure 4). As practice time increases, the FC of the basal ganglia tends to decrease, which may be attributed to the development of automaticity in complex motor skills during the later stages of skill acquisition (Doyon et al., 2009; Ashby et al., 2010; Dahms et al., 2020), particularly in the chunking of well-practiced movement sequences (Penhune and Steele, 2012).

Our study did not find a significant correlation between the number of years of dance training and the FC. The absence of association could be explained by the fact that longer training duration does not necessarily imply consistently greater length, quantity, or intensity of practice, nor does it guarantee a higher level of specialization (see Supplementary Figure 1 for the dissociation between training duration and practice intensity). Training duration primarily reflects the cumulative impact of long-term training, rather than the level of performance attained. On the other hand, the amount of practice time per day or week may indicate the current stage of skilled learning for a dancer. Our findings support the notion that the intensity of dance training plays a more significant role than the duration of training in determining trait effects. Moreover, subcortical functional connections may serve as an inverse predictor of the intensity of dance training.

The negative correlations observed between basal ganglia connections and originality in DANCE provide further support to the notion that embodied-based skill learning facilitates the extended cortico-subcortical MNS, which enables the generation of creative thinking with less motor and cognitive gating demands (Figure 5; Badre and Nee, 2018). The basal ganglia, dlPFC, and cerebellum are known to be neural substrates of novelty-based creative thinking (Khalil and Moustafa, 2022). The basal ganglia, in particular, are crucial in action selection for novelty detection, storage of motor information, and habit learning through repeated practice (Foerde and Shohamy, 2011; Penhune and Steele, 2012; Khalil and Moustafa, 2022).

In the integrated network/system, the basal ganglia, cerebellum, and thalamus are recognized as sensorimotor areas, while the dlPFC is considered a domain-general executive control area (Brown and Kim, 2021). The dlPFC critically subserves top-down executive control, regulating the flow of information (sensory, motor, and cognitive) through gating-like mechanisms and regulating the propagation of information across circuits (Badre and Nee, 2018; Tsuda et al., 2020). The negative correlation between originality and basal ganglia (GP and putamen) connections to the dlPFC in DANCE implies that dancers are not burdened by effortful sensory-motor and contextual control, allowing them to generate innovative movement ideas (Figures 5A, B; Shamay-Tsoory et al., 2011; Badre and Nee, 2018). The weakened connection with the right dlPFC in dancers may reflect weaker inhibition control of dance movements performed under unexpected environmental conditions to free their minds from well-established motor behavior (Shamay-Tsoory et al., 2011; Sainburg, 2016).

The present study provides additional evidence that the extended cortico-subcortical MNS is crucial to creativity, particularly originality, among dancers. Although the ATTA used mainly probes visual creativity, not motor creativity, the correlation with the integrated cortico-cerebellar-basal ganglia system implies the importance of embodied sensorimotor experiences in dancers. The embodied cognitive processes (e.g., creative thinking) in dancers may be grounded in sensorimotor experiences. Dance training involves a significant amount of multi-modal and cross-modal learning and sensorimotor processing. Intensive dance training may thus translate into enhanced novelty-based creative thinking in both the motor and visual domains (Fink et al., 2009; Palmiero et al., 2019). The efficiency with which these processes are executed can be attributed to attenuated cognitive-motor interactions, as evidenced by the high functionality of cortical-subcortical hierarchical organization (Caligiore et al., 2017).

### 4.5. Points of further consideration

A few points need consideration. Firstly, while the ReHo analysis is a useful tool for identifying important regions in the brain, its use alone may be limited for discussing long-range functional connectivity. To address this, besides our hierarchical approach, a network-based approach could be used in future research. Secondly, since the local and global functional integration of resting-state networks may be linked to specific cognitive abilities (Alavash et al., 2015), multiple cognitive tasks and psychological measures may be required to better understand the associations between mental abilities and brain neurodynamics at different spatial scales (local or global). Finally, it is quite common within the artistic education system in Taiwan for students to be exposed to multiple artistic disciplines from an early age. It is important to mention that none of the participants in both DANCE and CON reported having received any special training in sports. Taking into consideration the aforementioned information, we recognize that the potential influence of other art specialties, apart from dance, may be minimal, and the impact of sports training may be even smaller. Nevertheless, it remains challenging to entirely discount the potential effects of other art and sports, besides dance, on perception-action coupling.

## 5. Conclusion

Long-term dance training sculpts the brain of a dancer in a domain-specific way, as revealed by our hierarchical combination of ReHo and seed-based FC analysis. This analysis uncovered a neural “fingerprint” specific to highly trained dancers, indicating trait effects of dance training on neurodynamics. The strengthened connections observed in the resting state of dancers’ eMNS may contribute to their preparation for skilled performance. This is supported by the well-functioning cortical-subcortical hierarchical organization of the eMNS.

## Data availability statement

The raw data supporting the conclusions of this article will be made available by the authors, without undue reservation.

## Ethics statement

The studies involving human participants were reviewed and approved by the Institutional Review Board of Taipei Veterans General Hospital. The patients/participants provided their written informed consent to participate in this study.

## Author contributions

C-JY: conceptualization, investigation, formal analysis, validation, visualization, methodology, writing—original draft, and writing—review and editing. H-YY: resources and funding acquisition. T-YH: investigation. C-HS: investigation. T-CY: methodology and funding acquisition. L-FC: conceptualization, methodology, and funding acquisition. J-CH: conceptualization, resources, funding acquisition, project administration, methodology, supervision, and writing—review and editing. All authors contributed to the article and approved the submitted version.

## References

[B1] Alavash M. Doebler P. Holling H. Thiel C. M. Giessing C. (2015). Is functional integration of resting state brain networks an unspecific biomarker for working memory performance? *Neuroimage* 108 182–193. 10.1016/j.neuroimage.2014.12.046 25536495

[B2] Althuizen N. Wierenga B. Rossiter J. (2010). The validity of two brief measures of creative ability. *Creat. Res. J.* 22 53–61. 10.1080/10400410903579577

[B3] Ashburner J. (2007). A fast diffeomorphic image registration algorithm. *Neuroimage* 38 95–113. 10.1016/j.neuroimage.2007.07.007 17761438

[B4] Ashby F. G. Turner B. O. Horvitz J. C. (2010). Cortical and basal ganglia contributions to habit learning and automaticity. *Trends Cogn. Sci.* 14 208–215. 10.1016/j.tics.2010.02.001 20207189 PMC2862890

[B5] Bachrach A. Jola C. Pallier C. (2016). Neuronal bases of structural coherence in contemporary dance observation. *Neuroimage* 124(Pt A), 464–472. 10.1016/j.neuroimage.2015.08.072 26348557

[B6] Badre D. Nee D. E. (2018). Frontal cortex and the hierarchical control of behavior. *Trends Cogn. Sci.* 22 170–188. 10.1016/j.tics.2017.11.005 29229206 PMC5841250

[B7] Barsalou L. W. (2008). Grounded cognition. *Annu. Rev. Psychol.* 59 617–645. 10.1146/annurev.psych.59.103006.093639 17705682

[B8] Bläsing B. Calvo-Merino B. Cross E. S. Jola C. Honisch J. Stevens C. J. (2012). Neurocognitive control in dance perception and performance. *Acta Psychol.* 139 300–308. 10.1016/j.actpsy.2011.12.005 22305351

[B9] Bläsing B. Schack T. (2012). Mental representation of spatial movement parameters in dance. *Spatial Cogn. Comput.* 12 111–132. 10.1080/13875868.2011.626095

[B10] Boccia M. Piccardi L. Palermo L. Nori R. Palmiero M. (2015). Where do bright ideas occur in our brain? Meta-analytic evidence from neuroimaging studies of domain-specific creativity. *Front. Psychol.* 6:1195. 10.3389/fpsyg.2015.01195 26322002 PMC4531218

[B11] Bonini L. (2017). The extended mirror neuron network: anatomy, origin, and functions. *Neuroscientist* 23 56–67. 10.1177/1073858415626400 26747293

[B12] Bonini L. Rotunno C. Arcuri E. Gallese V. (2022). Mirror neurons 30 years later: implications and applications. *Trends Cogn. Sci.* 26 767–781. 10.1016/j.tics.2022.06.003 35803832

[B13] Brefczynski-Lewis J. A. Lutz A. Schaefer H. S. Levinson D. B. Davidson R. J. (2007). Neural correlates of attentional expertise in long-term meditation practitioners. *Proc. Natl. Acad. Sci. U.S.A.* 104 11483–11488. 10.1073/pnas.0606552104 17596341 PMC1903340

[B14] Brown S. Kim E. (2021). The neural basis of creative production: a cross-modal ALE meta-analysis. *Open Psychol.* 3 103–132. 10.1515/psych-2020-0114

[B15] Brown S. Martinez M. J. Parsons L. M. (2006). The neural basis of human dance. *Cereb. Cortex* 16 1157–1167. 10.1093/cercor/bhj057 16221923

[B16] Burzynska A. Z. Finc K. Taylor B. K. Knecht A. M. Kramer A. F. (2017). The dancing brain: structural and functional signatures of expert dance training. *Front. Hum. Neurosci.* 11:566. 10.3389/fnhum.2017.00566 29230170 PMC5711858

[B17] Butera C. Aziz-Zadeh L. (2022). “Mirror neurons and social implications for the classroom,” in *Movement matters: how embodied cognition informs teaching and learning*, eds Macrine S. L. Fugate J. M. B. (Cambridge, MA: The MIT Press).

[B18] Cahn B. R. Polich J. (2006). Meditation states and traits: EEG, ERP, and neuroimaging studies. *Psychol. Bull.* 132 180–211. 10.1037/0033-2909.132.2.180 16536641

[B19] Caligiore D. Arbib M. A. Miall R. C. Baldassarre G. (2019). The super-learning hypothesis: integrating learning processes across cortex, cerebellum and basal ganglia. *Neurosci. Biobehav. Rev.* 100 19–34. 10.1016/j.neubiorev.2019.02.008 30790636

[B20] Caligiore D. Pezzulo G. Baldassarre G. Bostan A. C. Strick P. L. Doya K. (2017). Consensus paper: towards a systems-level view of cerebellar function: the interplay between cerebellum, basal ganglia, and cortex. *Cerebellum* 16 203–229. 10.1007/s12311-016-0763-3 26873754 PMC5243918

[B21] Caligiore D. Pezzulo G. Miall R. C. Baldassarre G. (2013). The contribution of brain sub-cortical loops in the expression and acquisition of action understanding abilities. *Neurosci. Biobehav. Rev.* 37(10 Pt 2), 2504–2515. 10.1016/j.neubiorev.2013.07.016 23911926 PMC3878436

[B22] Calvo-Merino B. Ehrenberg S. Leung D. Haggard P. (2010). Experts see it all: configural effects in action observation. *Psychol. Res.* 74 400–406. 10.1007/s00426-009-0262-y 19856185

[B23] Calvo-Merino B. Glaser D. E. Grezes J. Passingham R. E. Haggard P. (2005). Action observation and acquired motor skills: an FMRI study with expert dancers. *Cereb. Cortex* 15 1243–1249. 10.1093/cercor/bhi007 15616133

[B24] Caramazza A. Anzellotti S. Strnad L. Lingnau A. (2014). Embodied cognition and mirror neurons: a critical assessment. *Annu. Rev. Neurosci.* 37 1–15. 10.1146/annurev-neuro-071013-013950 25032490

[B25] Carr L. Iacoboni M. Dubeau M. C. Mazziotta J. C. Lenzi G. L. (2003). Neural mechanisms of empathy in humans: a relay from neural systems for imitation to limbic areas. *Proc. Natl. Acad. Sci. U.S.A.* 100 5497–5502. 10.1073/pnas.0935845100 12682281 PMC154373

[B26] Caspers S. Zilles K. Laird A. R. Eickhoff S. B. (2010). ALE meta-analysis of action observation and imitation in the human brain. *Neuroimage* 50 1148–1167. 10.1016/j.neuroimage.2009.12.112 20056149 PMC4981639

[B27] Cattaneo L. Rizzolatti G. (2009). The mirror neuron system. *Arch. Neurol.* 66 557–560. 10.1001/archneurol.2009.41 19433654

[B28] Chen C.-Y. (2006). *Abbreviated torrance test for adults manual in Chinese version.* Taipei: Psychology Publisher.

[B29] Chen T.-T. Wang K.-P. Huang C.-J. Hung T.-M. (2022). Nonlinear refinement of functional brain connectivity in golf players of different skill levels. *Sci. Rep.* 12:2365. 10.1038/s41598-022-06161-3 35149719 PMC8837743

[B30] Chen Y.-H. Chang C.-Y. Huang S.-K. Yen N.-S. (2020). Nonlinear engagement of action observation network underlying action anticipation in players with different levels of expertise. *Hum. Brain Mapp.* 41 5199–5214. 10.1002/hbm.25186 32845066 PMC7670634

[B31] Christensen J. F. Cela-Conde C. J. Gomila A. (2017). Not all about sex: neural and biobehavioral functions of human dance. *Ann. N. Y. Acad. Sci.* 1400 8–32. 10.1111/nyas.13420 28787539

[B32] Christoff K. Irving Z. C. Fox K. C. Spreng R. N. Andrews-Hanna J. R. (2016). Mind-wandering as spontaneous thought: a dynamic framework. *Nat. Rev. Neurosci.* 17 718–731. 10.1038/nrn.2016.113 27654862

[B33] Clark A. (2016). *Surfing uncertainty: prediction, action, and the embodied mind.* New York, NY: Oxford University Press.

[B34] Cross E. S. Hamilton A. F. Grafton S. T. (2006). Building a motor simulation de novo: observation of dance by dancers. *Neuroimage* 31 1257–1267. 10.1016/j.neuroimage.2006.01.033 16530429 PMC1821082

[B35] Cross E. S. Kraemer D. J. Hamilton A. F. Kelley W. M. Grafton S. T. (2009). Sensitivity of the action observation network to physical and observational learning. *Cereb. Cortex* 19 315–326. 10.1093/cercor/bhn083 18515297 PMC2638791

[B36] Dahms C. Brodoehl S. Witte O. W. Klingner C. M. (2020). The importance of different learning stages for motor sequence learning after stroke. *Hum. Brain Mapp.* 41 270–286. 10.1002/hbm.24793 31520506 PMC7268039

[B37] Dayan E. Cohen L. G. (2011). Neuroplasticity subserving motor skill learning. *Neuron* 72 443–454. 10.1016/j.neuron.2011.10.008 22078504 PMC3217208

[B38] Deng L. Sun J. Cheng L. Tong S. (2016). Characterizing dynamic local functional connectivity in the human brain. *Sci. Rep.* 6:26976. 10.1038/srep26976 27231194 PMC4882585

[B39] Dong M. Qin W. Zhao L. Yang X. Yuan K. Zeng F. (2014). Expertise modulates local regional homogeneity of spontaneous brain activity in the resting brain: an fMRI study using the model of skilled acupuncturists. *Hum. Brain Mapp.* 35 1074–1084. 10.1002/hbm.22235 23633412 PMC6869809

[B40] Doyon J. Bellec P. Amsel R. Penhune V. Monchi O. Carrier J. (2009). Contributions of the basal ganglia and functionally related brain structures to motor learning. *Behav. Brain Res.* 199 61–75. 10.1016/j.bbr.2008.11.012 19061920

[B41] Doyon J. Penhune V. Ungerleider L. G. (2003). Distinct contribution of the cortico-striatal and cortico-cerebellar systems to motor skill learning. *Neuropsychologia* 41 252–262. 10.1016/s0028-3932(02)00158-6 12457751

[B42] Errante A. Fogassi L. (2020). Activation of cerebellum and basal ganglia during the observation and execution of manipulative actions. *Sci. Rep.* 10:12008. 10.1038/s41598-020-68928-w 32686738 PMC7371896

[B43] Fink A. Graif B. Neubauer A. C. (2009). Brain correlates underlying creative thinking: EEG alpha activity in professional vs. novice dancers. *Neuroimage* 46 854–862. 10.1016/j.neuroimage.2009.02.036 19269335

[B44] Fink A. Woschnjak S. (2011). Creativity and personality in professional dancers. *Pers. Individ. Differ.* 51 754–758. 10.1016/j.paid.2011.06.024

[B45] Foerde K. Shohamy D. (2011). The role of the basal ganglia in learning and memory: insight from Parkinson’s disease. *Neurobiol. Learn. Mem.* 96 624–636. 10.1016/j.nlm.2011.08.006 21945835 PMC3772079

[B46] Friston K. J. Williams S. Howard R. Frackowiak R. S. Turner R. (1996). Movement-related effects in fMRI time-series. *Magn. Reson. Med.* 35 346–355. 10.1002/mrm.1910350312 8699946

[B47] Fugate J. M. B. Macrine S. L. Cipriano C. (2018). The role of embodied cognition for transforming learning. *Int. J. Sch. Educ. Psychol.* 7 274–288. 10.1080/21683603.2018.1443856

[B48] Gonen-Yaacovi G. de Souza L. C. Levy R. Urbanski M. Josse G. Volle E. (2013). Rostral and caudal prefrontal contribution to creativity: a meta-analysis of functional imaging data. *Front. Hum. Neurosci.* 7:465. 10.3389/fnhum.2013.00465 23966927 PMC3743130

[B49] Goto A. (2022). Synaptic plasticity during systems memory consolidation. *Neurosci. Res.* 183 1–6. 10.1016/j.neures.2022.05.008 35667493

[B50] Grossberg S. (2016). “Neural dynamics of the basal ganglia during perceptual, cognitive, and motor learning and gating,” in *The basal ganglia: novel perspectives on motor and cognitive functions*, ed. Soghomonian J.-J. (Cham: Springer International Publishing), 457–512.

[B51] Gujing L. Hui H. Xin L. Lirong Z. Yutong Y. Guofeng Y. (2019). Increased insular connectivity and enhanced empathic ability associated with dance/music training. *Neural Plast.* 2019:9693109. 10.1155/2019/9693109 31198419 PMC6526550

[B52] Habas C. Kamdar N. Nguyen D. Prater K. Beckmann C. F. Menon V. (2009). Distinct cerebellar contributions to intrinsic connectivity networks. *J. Neurosci.* 29 8586–8594. 10.1523/JNEUROSCI.1868-09.2009 19571149 PMC2742620

[B53] Haber S. N. (2003). The primate basal ganglia: parallel and integrative networks. *J. Chem. Neuroanat.* 26 317–330. 10.1016/j.jchemneu.2003.10.003 14729134

[B54] Hanggi J. Koeneke S. Bezzola L. Jancke L. (2010). Structural neuroplasticity in the sensorimotor network of professional female ballet dancers. *Hum. Brain Mapp.* 31 1196–1206. 10.1002/hbm.20928 20024944 PMC6870845

[B55] Hardwick R. M. Rottschy C. Miall R. C. Eickhoff S. B. (2013). A quantitative meta-analysis and review of motor learning in the human brain. *Neuroimage* 67 283–297. 10.1016/j.neuroimage.2012.11.020 23194819 PMC3555187

[B56] Harmelech T. Preminger S. Wertman E. Malach R. (2013). The day-after effect: long term, Hebbian-like restructuring of resting-state fMRI patterns induced by a single epoch of cortical activation. *J. Neurosci.* 33 9488–9497. 10.1523/JNEUROSCI.5911-12.2013 23719815 PMC6618560

[B57] Hickok G. Houde J. Rong F. (2011). Sensorimotor integration in speech processing: computational basis and neural organization. *Neuron* 69 407–422. 10.1016/j.neuron.2011.01.019 21315253 PMC3057382

[B58] Hikosaka O. Nakamura K. Sakai K. Nakahara H. (2002). Central mechanisms of motor skill learning. *Curr. Opin. Neurobiol.* 12 217–222. 10.1016/s0959-4388(02)00307-0 12015240

[B59] Iacoboni M. (2005). Neural mechanisms of imitation. *Curr. Opin. Neurobiol.* 15 632–637. 10.1016/j.conb.2005.10.010 16271461

[B60] Karlinsky A. Zentgraf K. Hodges N. J. (2017). Action-skilled observation: issues for the study of sport expertise and the brain. *Prog. Brain Res.* 234 263–289. 10.1016/bs.pbr.2017.08.009 29031467

[B61] Karpati F. J. Giacosa C. Foster N. E. Penhune V. B. Hyde K. L. (2015). Dance and the brain: a review. *Ann. N. Y. Acad. Sci.* 1337 140–146. 10.1111/nyas.12632 25773628

[B62] Keysers C. Paracampo R. Gazzola V. (2018). What neuromodulation and lesion studies tell us about the function of the mirror neuron system and embodied cognition. *Curr. Opin. Psychol.* 24 35–40. 10.1016/j.copsyc.2018.04.001 29734039 PMC6173305

[B63] Khalil R. Moustafa A. A. (2022). A neurocomputational model of creative processes. *Neurosci. Biobehav. Rev.* 137:104656. 10.1016/j.neubiorev.2022.104656 35430189

[B64] Kharkhurin A. V. (2010). Sociocultural differences in the relationship between bilingualism and creative potential. *J. Cross Cult. Psychol.* 41 776–783. 10.1177/0022022110361777

[B65] Kharkhurin A. V. Samadpour Motalleebi S. N. (2008). The impact of culture on the creative potential of american, russian, and iranian college students. *Crea. Res. J.* 20 404–411. 10.1080/10400410802391835

[B66] Kim S.-G. Knosche T. R. (2017). Resting state functional connectivity of the ventral auditory pathway in musicians with absolute pitch. *Hum. Brain Mapp.* 38 3899–3916. 10.1002/hbm.23637 28481006 PMC6866733

[B67] Koziol L. F. Budding D. E. Chidekel D. (2012). From movement to thought: executive function, embodied cognition, and the cerebellum. *Cerebellum* 11 505–525. 10.1007/s12311-011-0321-y 22068584

[B68] Lehericy S. Benali H. Van de Moortele P. F. Pelegrini-Issac M. Waechter T. Ugurbil K. (2005). Distinct basal ganglia territories are engaged in early and advanced motor sequence learning. *Proc. Natl. Acad. Sci. U.S.A.* 102 12566–12571. 10.1073/pnas.0502762102 16107540 PMC1194910

[B69] Levy B. J. Wagner A. D. (2011). Cognitive control and right ventrolateral prefrontal cortex: reflexive reorienting, motor inhibition, and action updating. *Ann. N. Y. Acad. Sci.* 1224 40–62. 10.1111/j.1749-6632.2011.05958.x 21486295 PMC3079823

[B70] Lewis C. M. Baldassarre A. Committeri G. Romani G. L. Corbetta M. (2009). Learning sculpts the spontaneous activity of the resting human brain. *Proc. Natl. Acad. Sci. U.S.A.* 106 17558–17563. 10.1073/pnas.0902455106 19805061 PMC2762683

[B71] Li G. He H. Huang M. Zhang X. Lu J. Lai Y. (2015). Identifying enhanced cortico-basal ganglia loops associated with prolonged dance training. *Sci. Rep.* 5:10271. 10.1038/srep10271 26035693 PMC4649913

[B72] Lin C.-S. Liu Y. Huang W.-Y. Lu C.-F. Teng S. Ju T.-C. (2013). Sculpting the Intrinsic modular organization of spontaneous brain activity by art. *PLoS One* 8:e66761. 10.1371/journal.pone.0066761 23840527 PMC3694132

[B73] Lu Y. Zhao Q. Wang Y. Zhou C. (2018). Ballroom dancing promotes neural activity in the sensorimotor system: a resting-state fMRI study. *Neural Plast.* 2018:2024835. 10.1155/2018/2024835 29853838 PMC5944238

[B74] Luo C. Tu S. Peng Y. Gao S. Li J. Dong L. (2014). Long-term effects of musical training and functional plasticity in salience system. *Neural Plast.* 2014:180138. 10.1155/2014/180138 25478236 PMC4247966

[B75] Macpherson T. Hikida T. (2019). Role of basal ganglia neurocircuitry in the pathology of psychiatric disorders. *Psychiatry Clin. Neurosci.* 73 289–301. 10.1111/pcn.12830 30734985

[B76] Macrine S. L. Fugate J. M. B. (2020). *Embodied cognition.* Oxford: Oxford University Press.

[B77] Macrine S. L. Fugate J. M. B. (2021). Translating Embodied Cognition for Embodied Learning in the Classroom. *Front. Educ.* 6:712626. 10.3389/feduc.2021.712626

[B78] Maldjian J. A. Laurienti P. J. Kraft R. A. Burdette J. H. (2003). An automated method for neuroanatomic and cytoarchitectonic atlas-based interrogation of fMRI data sets. *Neuroimage* 19 1233–1239. 10.1016/s1053-8119(03)00169-1 12880848

[B79] Miall R. C. (2003). Connecting mirror neurons and forward models. *Neuroreport* 14 2135–2137. 10.1097/00001756-200312020-00001 14625435

[B80] Molenberghs P. Cunnington R. Mattingley J. B. (2012). Brain regions with mirror properties: a meta-analysis of 125 human fMRI studies. *Neurosci. Biobehav. Rev.* 36 341–349. 10.1016/j.neubiorev.2011.07.004 21782846

[B81] Molnar-Szakacs I. Iacoboni M. Koski L. Mazziotta J. C. (2005). Functional segregation within pars opercularis of the inferior frontal gyrus: evidence from fMRI studies of imitation and action observation. *Cereb. Cortex* 15 986–994. 10.1093/cercor/bhh199 15513929

[B82] Murphy K. Birn R. M. Handwerker D. A. Jones T. B. Bandettini P. A. (2009). The impact of global signal regression on resting state correlations: are anti-correlated networks introduced? *Neuroimage* 44 893–905. 10.1016/j.neuroimage.2008.09.036 18976716 PMC2750906

[B83] Mutha P. K. Haaland K. Y. Sainburg R. L. (2012). The effects of brain lateralization on motor control and adaptation. *J. Mot. Behav.* 44 455–469. 10.1080/00222895.2012.747482 23237468 PMC3549328

[B84] Nordin S. M. Cumming J. (2005). Professional dancers describe their imagery: where, when, what, why, and how. *Sport Psychol.* 19 395–416. 10.1123/tsp.19.4.395

[B85] Novembre G. Keller P. E. (2014). A conceptual review on action-perception coupling in the musicians’ brain: what is it good for? *Front. Hum. Neurosci.* 8:603. 10.3389/fnhum.2014.00603 25191246 PMC4139714

[B86] Oldfield R. C. (1971). The assessment and analysis of handedness: the Edinburgh inventory. *Neuropsychologia* 9 97–113. 10.1016/0028-3932(71)90067-4 5146491

[B87] Palmiero M. Giulianella L. Guariglia P. Boccia M. D’Amico S. Piccardi L. (2019). The dancers’ Visuospatial body map explains their enhanced divergence in the production of motor forms: evidence in the early development. *Front. Psychol.* 10:768. 10.3389/fpsyg.2019.00768 31024403 PMC6467967

[B88] Penhune V. B. Steele C. J. (2012). Parallel contributions of cerebellar, striatal and M1 mechanisms to motor sequence learning. *Behav. Brain Res.* 226 579–591. 10.1016/j.bbr.2011.09.044 22004979

[B89] Pineda J. A. (2008). Sensorimotor cortex as a critical component of an ‘extended’ mirror neuron system: does it solve the development, correspondence, and control problems in mirroring? *Behav. Brain Funct.* 4:47. 10.1186/1744-9081-4-47 18928566 PMC2577683

[B90] Pu M. Heleven E. Delplanque J. Gibert N. Ma Q. Funghi G. (2020). The posterior cerebellum supports the explicit sequence learning linked to trait attribution. *Cogn. Affect. Behav. Neurosci.* 20 798–815. 10.3758/s13415-020-00803-7 32495270 PMC7395039

[B91] Ramsey R. Kaplan D. M. Cross E. S. (2021). Watch and learn: the cognitive neuroscience of learning from others’. Actions. *Trends Neurosci.* 44 478–491. 10.1016/j.tins.2021.01.007 33637286

[B92] Rizzolatti G. Craighero L. (2004). The mirror-neuron system. *Annu. Rev. Neurosci.* 27 169–192. 10.1146/annurev.neuro.27.070203.144230 15217330

[B93] Rizzolatti G. Fogassi L. Gallese V. (2001). Neurophysiological mechanisms underlying the understanding and imitation of action. *Nat. Rev. Neurosci.* 2 661–670. 10.1038/35090060 11533734

[B94] Saad Z. S. Gotts S. J. Murphy K. Chen G. Jo H. J. Martin A. (2012). Trouble at rest: how correlation patterns and group differences become distorted after global signal regression. *Brain Connect.* 2 25–32. 10.1089/brain.2012.0080 22432927 PMC3484684

[B95] Sainburg R. L. (2016). “Laterality of basic motor control mechanisms,” in *Laterality in sports: Theories and applications*, eds Hagemann N. Strauss B. MacMahon C. (San Diego, CA: Academic Press), 155–177.

[B96] Sevdalis V. Keller P. E. (2011). Captured by motion: dance, action understanding, and social cognition. *Brain Cogn.* 77 231–236. 10.1016/j.bandc.2011.08.005 21880410

[B97] Shamay-Tsoory S. G. Adler N. Aharon-Peretz J. Perry D. Mayseless N. (2011). The origins of originality: the neural bases of creative thinking and originality. *Neuropsychologia* 49 178–185. 10.1016/j.neuropsychologia.2010.11.020 21126528

[B98] Shen T. Lai J.-C. (2014). Exploring the relationship between creative test of ATTA and the thinking of creative works. *Procedia Soc. Behav. Sci.* 112 557–566. 10.1016/j.sbspro.2014.01.1202

[B99] Siman-Tov T. Granot R. Y. Shany O. Singer N. Hendler T. Gordon C. R. (2019). Is there a prediction network? Meta-analytic evidence for a cortical-subcortical network likely subserving prediction. *Neurosci. Biobehav. Rev.* 105 262–275. 10.1016/j.neubiorev.2019.08.012 31437478

[B100] Starowicz-Filip A. Prochwicz K. Klosowska J. Chrobak A. A. Myszka A. Betkowska-Korpala B. (2021). Cerebellar functional lateralization from the perspective of clinical neuropsychology. *Front. Psychol.* 12:775308. 10.3389/fpsyg.2021.775308 34955995 PMC8703197

[B101] Sunavsky A. Poppenk J. (2020). Neuroimaging predictors of creativity in healthy adults. *Neuroimage* 206:116292. 10.1016/j.neuroimage.2019.116292 31654758

[B102] Tian L. Ren J. Zang Y. (2012). Regional homogeneity of resting state fMRI signals predicts stop signal task performance. *Neuroimage* 60 539–544. 10.1016/j.neuroimage.2011.11.098 22178814

[B103] Tsuda B. Tye K. M. Siegelmann H. T. Sejnowski T. J. (2020). A modeling framework for adaptive lifelong learning with transfer and savings through gating in the prefrontal cortex. *Proc. Natl. Acad. Sci. U.S.A.* 117 29872–29882. 10.1073/pnas.2009591117 33154155 PMC7703668

[B104] Turella L. Wurm M. F. Tucciarelli R. Lingnau A. (2013). Expertise in action observation: recent neuroimaging findings and future perspectives. *Front. Hum. Neurosci.* 7:637. 10.3389/fnhum.2013.00637 24137118 PMC3797401

[B105] Tzourio-Mazoyer N. Landeau B. Papathanassiou D. Crivello F. Etard O. Delcroix N. (2002). Automated anatomical labeling of activations in SPM using a macroscopic anatomical parcellation of the MNI MRI single-subject brain. *Neuroimage* 15 273–289. 10.1006/nimg.2001.0978 11771995

[B106] Udden J. Bahlmann J. (2012). A rostro-caudal gradient of structured sequence processing in the left inferior frontal gyrus. *Philos. Trans. R. Soc. Lond. B Biol. Sci.* 367 2023–2032. 10.1098/rstb.2012.0009 22688637 PMC3367683

[B107] Uddin L. Q. Kinnison J. Pessoa L. Anderson M. L. (2014). Beyond the tripartite cognition-emotion-interoception model of the human insular cortex. *J. Cogn. Neurosci.* 26 16–27. 10.1162/jocn_a_00462 23937691 PMC4074004

[B108] Walther S. Friederich H. C. Stippich C. Weisbrod M. Kaiser S. (2011). Response inhibition or salience detection in the right ventrolateral prefrontal cortex? *Neuroreport* 22 778–782. 10.1097/WNR.0b013e32834af670 21876462

[B109] Weissenbacher A. Kasess C. Gerstl F. Lanzenberger R. Moser E. Windischberger C. (2009). Correlations and anticorrelations in resting-state functional connectivity MRI: a quantitative comparison of preprocessing strategies. *Neuroimage* 47 1408–1416. 10.1016/j.neuroimage.2009.05.005 19442749

[B110] Wolpert D. M. Doya K. Kawato M. (2003). A unifying computational framework for motor control and social interaction. *Philos. Trans. R. Soc. Lond. B Biol. Sci.* 358 593–602. 10.1098/rstb.2002.1238 12689384 PMC1693134

[B111] Worbe Y. Malherbe C. Hartmann A. Pelegrini-Issac M. Messe A. Vidailhet M. (2012). Functional immaturity of cortico-basal ganglia networks in Gilles de la Tourette syndrome. *Brain* 135(Pt 6), 1937–1946. 10.1093/brain/aws056 22434213

[B112] Wu T.-H. Tu C.-H. Chao H.-T. Li W.-C. Low I. Chuang C.-Y. (2016). Dynamic changes of functional pain connectome in women with primary dysmenorrhea. *Sci. Rep.* 6:24543. 10.1038/srep24543 27089970 PMC4835697

[B113] Yan C.-G. Wang X.-D. Zuo X.-N. Zang Y.-F. (2016). DPABI: data processing & analysis for (resting-state) brain imaging. *Neuroinformatics* 14 339–351. 10.1007/s12021-016-9299-4 27075850

[B114] Yan F.-X. Wu C.-W. Cheng S.-Y. Lim K.-E. Hsu Y.-Y. Liu H.-L. (2013). Resting-state functional magnetic resonance imaging analysis with seed definition constrained by regional homogeneity. *Brain Connect.* 3 438–449. 10.1089/brain.2013.0164 23802999

[B115] Yarrow K. Brown P. Krakauer J. W. (2009). Inside the brain of an elite athlete: the neural processes that support high achievement in sports. *Nat. Rev. Neurosci.* 10 585–596. 10.1038/nrn2672 19571792

[B116] Yin H. H. Knowlton B. J. (2006). The role of the basal ganglia in habit formation. *Nat. Rev. Neurosci.* 7 464–476. 10.1038/nrn1919 16715055

[B117] Zang Y. Jiang T. Lu Y. He Y. Tian L. (2004). Regional homogeneity approach to fMRI data analysis. *Neuroimage* 22 394–400. 10.1016/j.neuroimage.2003.12.030 15110032

[B118] Zardi A. Carlotti E. G. Pontremoli A. Morese R. (2021). Dancing in your head: an interdisciplinary review. *Front. Psychol.* 12:649121. 10.3389/fpsyg.2021.649121 34002113 PMC8123236

[B119] Zuo X.-N. Xu T. Jiang L. Yang Z. Cao X.-Y. He Y. (2013). Toward reliable characterization of functional homogeneity in the human brain: preprocessing, scan duration, imaging resolution and computational space. *Neuroimage* 65 374–386. 10.1016/j.neuroimage.2012.10.017 23085497 PMC3609711

