# Supplementary material for: Trait representation of embodied cognition in dancers pivoting on the extended mirror neuron system: a resting-state fMRI study
Source: Front Hum Neurosci. 2023 Jul 10;17:1173993. doi: 10.3389/fnhum.2023.1173993 (PMC10364845; doi:10.3389/fnhum.2023.1173993)
Supplement: Supplementary file 1 [file Image_1.pdf]

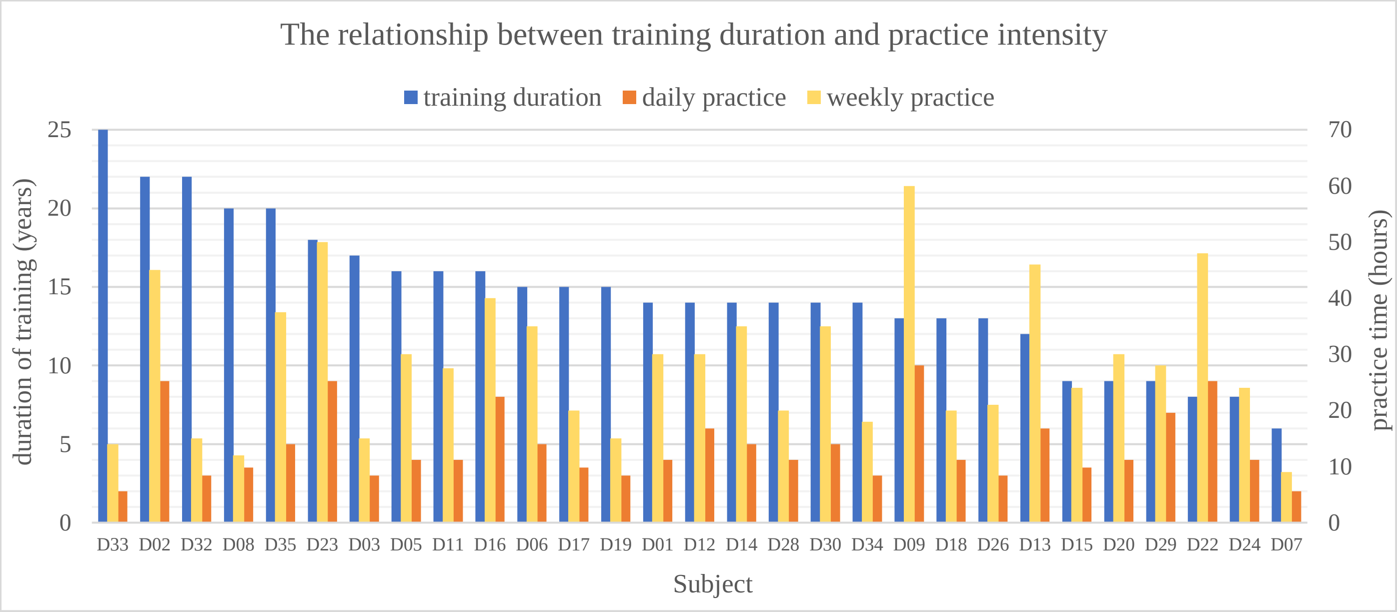

Supplementary Figure 1. The relationship between training duration and practice intensity. The dual-axis chart illustrates the dissociation between training duration (measured in years) and practice intensity (measured in hours per day or week) on distinct scales. The figure clearly demonstrates that a longer training duration does not necessarily correspond consistently to higher practice intensity. The x-axis represents each dancer in the current study. The left y-axis represents years of training duration, while the right y-axis represents hours of practice time per week. The blue bar graph represents years of dance training, arranged in a descending order. The yellow bar graph represents average practice time per week, while the orange bar graph represents average practice time per day.
